# HIV Prevalence among Aboriginal British Columbians

**DOI:** 10.1186/1477-7517-2-26

**Published:** 2005-12-24

**Authors:** Robert S Hogg, Steffanie Strathdee, Thomas Kerr, Evan Wood, Robert Remis

**Affiliations:** 1 BC Centre for Excellence in HIV/AIDS; 2 Department of Medicine, University of British Columbia, Vancouver, British Columbia, Canada; 3 Department of Health Care and Epidemiology, Faculty of Medicine, University of British Columbia, Vancouver, British Columbia, Canada; 4 Department of Family and Preventive Medicine, Division of International Health, University of California, San Diego School of Medicine, California; 5 Department of Public Health Sciences, Faculty of Medicine, University of Toronto, University of Toronto, Canada

## Abstract

**Context:**

There is considerable concern about the spread of HIV disease among Aboriginal peoples in British Columbia.

**Objective:**

To estimate the number of Aboriginal British Columbians infected with HIV.

**Design and setting:**

A population-based analysis of Aboriginal men and women in British Columbia, Canada from 1980 to 2001.

**Participants:**

Epidemic curves were fit for gay and bisexual men, injection drug users, men and women aged 15 to 49 years and persons over 50 years of age.

**Main outcome measures:**

HIV prevalence for the total Aboriginal population was modeled using the UNAIDS/WHO Estimation and Projection Package (EPP). Monte Carlo simulation was used to estimate potential number infected for select transmission group in 2001.

**Results:**

A total of 170,025 Aboriginals resided in British Columbia in 2001, of whom 69% were 15 years and older. Of these 1,691 (range 1,479 – 1,955) men and women aged 15 years and over were living with HIV with overall prevalence ranging from 1.26% to 1.66%. The majority of the persons infected were men. Injection drug users (range 1,202 – 1,744) and gay and bisexual men (range 145, 232) contributed the greatest number of infections. Few persons infected were from low risk populations.

**Conclusion:**

More than 1 in every 100 Aboriginals aged 15 years and over was living with HIV in 2001. Culturally appropriate approaches are needed to tailor effective HIV interventions to this community.

## Introduction

Aboriginal peoples have resided in British Columbia since the end of the last ice age 12,000 years ago [1]. Archaeological evidence suggests that these first peoples arrived here through successive migrations across a land bridge spanning the Bering Strait and then arrived here either along the coast [2] or through an interior passage left as the glacier sheets melted [3]. Three distinct cultural areas with many distinct cultures are prominent – the subartic in the Northeast, the plateau in the Southeast, and the Northwest Coast along the coast from the Queen Charlottes to the southern most tip of Vancouver Island. Prior to contact with Europeans, populations were quite large especially along the coast.

Contact with Europeans brought numerous infectious diseases that reduced the Aboriginal population in British Columbia by nearly two thirds. The total population continued to decrease until the 1920s and have since rebounded to sizes near pre-contact levels [4,5]. However, high levels of mortality persisted long after European contact, mainly due to infectious diseases [4,6]. Initially, disease like smallpox had a devastating affect on population size [4]. Later, Aboriginals where inflicted with diseases like tuberculosis that were endemic until quite recently [7]. In recent years, there has been increasing concern regarding the spread of HIV disease among Aboriginal peoples. HIV appears to be concentrated among injection drug users and gay and bisexual men [8-11]. Rates of HIV infection among pregnant women remain low, but are significantly higher than in the general population[12]. The purpose of this paper is to estimate the current number of Aboriginal British Columbians infected with HIV.

## Methods

Our estimates of HIV prevalence in the British Columbian Aboriginal population were based upon surveillance data. Aboriginals refer to persons who self-identify as – North American Indians, Métis, and Inuit. Also included are those that did not self-identify as Aboriginal, but who were registered under the Indian Act and/or were members of a band or First Nation [13].

HIV prevalence data from at risk populations were used to model HIV prevalence trends for adults and to calculate the number of new infections, AIDS cases and deaths from 1980 to 2001. Six adult Aboriginal population groups, aged 15 years and over, were modeled – gay and bisexual men, injection drug users in Greater Vancouver and the rest of the province, low-risk men and women aged 15 to 49 years, and low-risk persons aged 50 years and over. Low-risk refers to Aboriginal persons who based on current seroprevalence studies were less likely to acquire HIV than gay and bisexual men and injection drug users in this population. Gay and bisexual men were estimated to be 3% of the total Aboriginal male population [14]. The total injection drug user population in Greater Vancouver was based on capture-recapture estimates and for the rest of the province on estimates of clean needle distributed at needle exchanges and proportion of drug over deaths [15,16]. Based on estimates derived from the Vancouver Injection Drug Users Study (VIDUS) at least quarter of these injection drug users are of Aboriginal descent [8]. The population of injection drug users in the Greater Vancouver region was estimated to be 12,000 (95% CI: 10,000 – 15,000) and of similar magnitude outside the region as half the needles distributed at needle exchanges and overdose deaths occur outside of Greater Vancouver[16]. In total, there were likely 24,000 injection drug users in the province of which 6,000 were Aboriginal. Population estimates for low-risk persons aged 15 to 49 years and those laged 50 years and over were based intercensal estimates of Registered Indians produced by BC Vital Statistics and adjusted to the 2001 Census population of Aboriginals [16].

HIV prevalence estimates were obtained from a variety of serosurveillance and cohort studies. Estimates of HIV prevalence for the gay and bisexual men were obtained from published and unpublished estimates from two cohorts studies (Vancouver Lymphadenopathy AIDS Study and the Vanguard Study) and adjusted to estimates reported for the entire gay and bisexual male population in Vancouver and British Columbia [12,17]. Among injection drug users HIV prevalence estimates were obtained from published cohort and cross sectional studies. Annualized estimates of HIV prevalence from 1996 onwards were obtained from VIDUS[8,18]. Estimates prior to that date were obtained from serosurveillance studies of street-based populations and needle exchanges in Vancouver and Victoria[12]. HIV prevalence estimates of low-risk persons aged 15 to 49 years and 50 years and over were based on data obtained from pregnant women and men and women in alcohol rehabilitation[12,19,20].

Two scenarios were modelled based on varying assumptions relating to the size of the at-risk population and HIV prevalence (refer to Table 1). The low growth scenario assumed the at-risk gay and bisexual population to be 3% of the adult male population. The population of Aboriginal injection drug user in Greater Vancouver was assumed to be 3,000 or 25% of injection drug users in that region, the point estimate from the capture-recapture study[15]. Injection drug user population outside this region was also assumed to be 3,000 since half of the overdose deaths and clean needles distributed occur in this region. HIV prevalence in gay and bisexual men was assumed to be similar to the general gay and bisexual population. In injection drug users HIV prevalence was assumed to increase to 40% in Vancouver and to move-up to no more than 2% in other areas of the province. HIV prevalence in low-risk persons aged 15 to 49 years and 50 years and over was assumed to be zero.

**Table 1:** Input assumptions for total population and HIV prevalence for Aboriginal persons ages 15 and over, by scenario and group

**Category/ years**	**Gay and Bisexual men**	**Injection drug users**		15–49 years*		**50+**
**Year**		**Vancouver**	**Other**	**Men**	**Women**	
**Low growth scenario**						

**Population**						
2001	1,600	3,000	3,000	39,400	46,700	24,100
**HIV Prevalence**						
1986	12.6	1.2	1.2	0	0	0
1991	14.3	1.7	1.7	0	0	0
1996	15.0	6.0	2.0*	0	0	0
2001	19.0	38.0	2.0*	0	0	0

**High growth scenario**						

**Population**						
2001	1,600	4,250	3,250	38,700	45,900	24,100
**HIV Prevalence**						
1986	12.6	2.3	2.3	0	0	0
1991	14.3	1.7	1.7	0	0	0
1996	15.0	6.0	6.0*	0	0	0
2001	19.0	38.0	6.0*	.08	.10	0

* Values are assumed. Among 15 to 49 years olds posted rates are a third of published rates.

The high growth scenario assumed the gay and bisexual male population to be the same as in the low growth scenario. The Greater Vancouver Aboriginal injection drug user population was assumed to be 3,750 persons or 25% of the upper limit of the capture-recapture estimate of 15,000 [15]. A total of 500 Aboriginal injection drug users in Victoria were assumed to have the same HIV prevalence as in Greater Vancouver and added to the Greater Vancouver group[21]. Among the remaining 3,250 injection drug users from outside of Vancouver and Victoria, HIV prevalence was assumed to increase to 6% by 1996 [12]. HIV prevalence in low-risk women aged 15 to 49 years was assumed to increase to .08 by 2001 or a third of estimates obtained from studies of pregnant women and women in alcohol rehabilitation. HIV prevalence in low-risk men aged 15–49 was also assumed to increase and the number of HIV-infected persons aged 50 years and over was assumed to be zero.

HIV prevalence for the total Aboriginal population was modeled using the UNAIDS/WHO Estimation and Projection Package (EPP). In EPP, HIV prevalence time trends were estimated by fitting a simple epidemiological model to surveillance data[22]. Epidemic curves were fit for gay and bisexual men, injection drug users, men and women aged 15 to 49 years and persons over 65 years of age. Separate epidemic curves for these groups were then aggregated by EPP to find the best fitting curve that describes trends in HIV prevalence in the total adult Aboriginal over time.

Monte Carlo simulation methodology was used to estimate potential number infected for select transmission group in 2001. Input parameters for the model were taken from our low and high growth scenarios. The injection drug user group was collapsed into one category for these analyses. A total of 100,000 trials were completed to derive potential ranges in the number of injection drug users, gay and bisexual men, and low risk women in the population.

## Results

A total of 170,025 Aboriginals resided in the province in 2001, of whom 69% were 15 years and older. The majority of Aboriginals were women (51%). The median age for men and women was 26 and 28 years, respectively. North American Indians made-up the largest component of the population at 118,295 (70%), followed by Metis at 44,265 (26%), and Inuit at 800 (0.5%). The rest, 6660 (4%) were Aboriginals of other or multiple origin. The Greater Vancouver population was 36,855 with 10,440 living in the city. The population was also highly mobile with 46% of persons who were 5 years and older moving at least once in the past five years.

Table 1 outlines the at-risk population and HIV prevalence assumptions for Scenarios one and two. As outlined in this table the injection drug users and gay and bisexual male populations are relatively small in comparison to the low-risk populations aged 15 and over. In both scenarios HIV prevalence is highest in Greater Vancouver injection drug users and second highest among gay and bisexual men.

**Figure 1:**
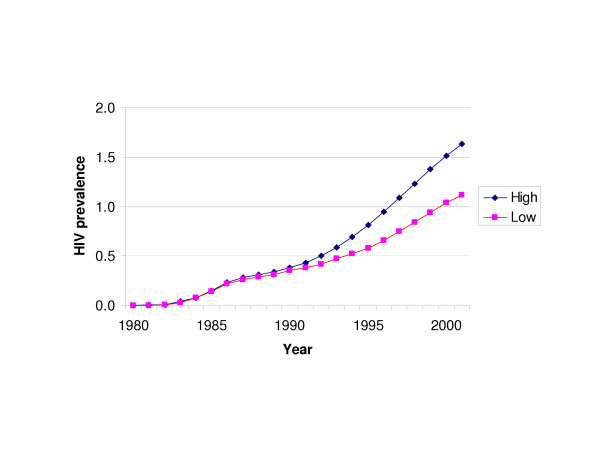
HIV prevalence among Aboriginal British Columbians by scenario, 1980 to 2001.

Figures 1 characterizes the trends in HIV prevalence in the low and high growth scenarios since 1980. As note shown here, HIV prevalence in the Aboriginal population has increased notably since 1980.

Table 2 provides estimates of prevalence and the number living with HIV by gender and transmission group in 2001. Estimates were derived from 100,000 Monte Carlo simulation trials, where the input parameters were based on the figures from the low and high growth scenarios produced for this study. Based on this analysis, a total of 1,691 (range 1,479 – 1,955) men and women were living with HIV at the end of 2001. Overall, 1.44% (range 1.26%, 1.66%) of the population 15 years and over was HIV-positive. The majority of the persons infected were men (55.3% ; range 55.6%, 54.8%). Injection drug users (1,458; range 1,202 – 1,744) and gay and bisexual men (186; range 145, 232) contributed the greatest number of infections. Few of the persons living with HIV were from low risk populations.

**Table 2:** Estimated prevalence and number of Aboriginal British Columbians living with HIV in 2001*

	**Monte Carlo Simulation**	
	
**Variable**	**Estimate**	**2.5 and 97.5th percentiles**
**Population aged 15 and above**	117,800	
		
**HIV prevalence**	1.44%	1.26% – 1.66%
**HIV infected**		
Males	935	822 – 1,072
Females	756	652 – 887
Total	1,691	1,479 – 1,955
		
**HIV infected by group**		
Gay and bisexual men	186	145 – 232
Injection drug users	1,458	1,202 – 1,744
Low-risk men 15–49 years	16	3 – 29
Low risk women 15–49 years	23	6 – 41
Low-risk population 50 years and over	6	5 – 7

* Derived from the results of 100,000 Monte Carlo simulation trials.

## Discussion

More than 1 in every 100 Aboriginal persons aged 15 years and over was living with HIV in 2001. Approximately a quarter to a third of all infections among Aboriginal peoples in Canada occurred in this province [23]. The rate of infection among Aboriginal British Columbians was approximately two times the rate for Canadian Aboriginals overall. The majority of new infections among Aboriginals occurred in injection drug users with the majority of these being concentrated in Greater Vancouver. However, our results point to increasing numbers of injection drug users being infected outside Greater Vancouver and considerable number of Aboriginal gay and bisexual men infected with HIV in this province.

The high prevalence of HIV occurring among British Columbian Aboriginals was mainly due to increases in new infections among injection drug users. In Vancouver, HIV prevalence among Aboriginal injection drug users has increased from less than 5% in early 1990s to approximately 40% in 2004 [24]. The prevalence of HIV among Aboriginal injection drug users was considerably higher than their non-Aboriginal counterparts; and half of the Aboriginal drug user population were women, which was a considerably higher proportion than in the non-Aboriginal population [8]. Risk factors for HIV acquisition appear to vary by gender as well. Among women, frequent speedball (combined cocaine and heroin) injection and going on binges of injection drug use were independent predictors of HIV seroconversion; while among men HIV seroconversion was associated with frequent speedball injection and cocaine injection [8,18]

The acquisition of HIV among Aboriginals was partially due to syringe sharing. Needle exchanges in British Columbia provide needles, needle cleaning supplies and condoms to injection drug users and sex trade workers. Although six millions needles are given out annually through this program, needle exchanges provide sterile equipment for only a small percentage of drug injection episodes – in Vancouver this was estimated to be as low as 10 to 20% [25]. Among injection drug users daily needle exchanges remains modest at approximately 40% at fixed sites and 50% at mobile sites [26]. Even with daily exchange approximately a third of participants were borrowing and lending needles. Although acquiring needles exclusively from the needle exchange attendance was independently associated with less sharing, persistent sharing was associated with difficulty accessing sterile needles, bingeing, and frequent cocaine injection [27].

Unprotected sex also played a role in the acquisition of new infections. There was considerable variability among injection drug users in condom use with sex trade clients and casual and regular sexual partners [8,18,28]. Based on VIDUS data the vast majority of men and women were sexually active, 72 and 92% respectively in the last six months prior to enrolment. The mean age of first sexual encounter was 15 years for either gender. Life time number of sexual partners was also high with over 20% of men and 50% of women having more than 100 sexual partners. Among men and women, condoms were generally not used with regular partners, half the time with casual partners, and 80% of the time with clients. Nine per cent of men reported having had sex with a man with condom use being 71% for anal intercourse and 60% for oral sex with clients. Among women sex trade workers vaginal intercourse was most common with condom usage being 82%. Seventy-five percent of these women used condoms during oral sex with clients. Aboriginal generally exhibit the same pattern of condom usage as non-Aboriginal persons in this cohort.

Our results point to a number of important policy implications and gaps in knowledge. If our estimates of injection drug users are correct then a considerable number of injection drug users are living outside of Greater Vancouver and likely do not have the same access to harm reduction services or have to travel further to get to them. Persons outside of Vancouver and Victoria are known to have limited access to drug rehabilitation program or to methadone. The effectiveness of needle exchanges in places like Campbell River, Chilliwack, Gibsons, Kamloops, Kelowna, Nanaimo, Powell River, Prince George, Prince Rupert, Quesnel, Surrey, and Veron is also not well characterized. Little is known about HIV prevalence of injection drug users at these sites, and whether reported rates are higher as or much lower than those observed in Vancouver and Victoria.

Although the population of Aboriginal gay and bisexual men is small it still accounts for a large proportion of HIV infections. Little is known about how much of male to male sexual activity is attributed to sex work. Future prevention work in this community needs to be targeted, even though these men are more likely to be more marginalized and in the sex trade than their non-Aboriginal gay and bisexual counterparts[9].

Finally, the spread of HIV to the general Aboriginal population is not well characterized. There is no conclusive evidence to suggestion that the increase in seroprevalence among pregnant women and low risk men is not directly attributable to injection drug use. We need to ensure that all Aboriginal pregnant women have access to HIV testing and to antenatal care. Currently, few Aboriginal women who have HIV-positive children are able to seek adequate care for themselves or their children[20]. Access to antiretroviral therapy among those infected needs to be improved. Aboriginal British Columbians are also not accessing treatment at the same rate as non-Aboriginals[29]. Differences in access are likely attributed to differences in living conditions, access to physicians, as well as the remoteness of some communities.

In conclusion, more than 1 in every 100 adults aged 15 years and over was living with HIV in 2001. The rate of infection observed in this study was greatly affected by the size of the injection drug user population and the spread of HIV into the general population. Governments and non-government organizations need to work together to ensure the funding of culturally appropriate HIV prevention programs.
